# Some Names That Mislead

**Published:** 1888-11

**Authors:** 


### *SOME NAMES THAT MISLEAD.*

The tuberose is no rose, but a species of olyanth.

Pompey's pillar had no historical connection with Pompey in any way.

Cleopatra's needle was not erected by the Egyptian Queen, nor in her honor.

Whalebone is not bone, and is said not to possess a single property of bone.

Turkish baths did not originate in Turkey, and are not baths, only heated chambers.

German silver was not invented in Germany, and does not contain a particle of silver.

Black lead is not lead at all, but a compound ot carbon and a small quantity of iron.

Brazilian grass never grew in Brazil, and is not grass; it is nothing but strips of palm-leaf.

Burgundy pitch is not pitch, and does not come from Burgundy; the greater part of it is resin and palm-oil.

Sealing wax does not contain a particle of wax, but is composed of Venice turpentine, shellac and cinnabar.

Catgut is made from the entrails of sheep.

Cuttle-bone is not bone, but a kind of chalk once inclosed in the fossil remains of extinct specimens of cuttle-fish.—*Medical Herald, Louisville.*

**In a St. Louis dissecting-room a subject without clavicles has been found, these bones being replaced by fibrous cords.**

